# Key microRNAs and hub genes associated with poor prognosis in lung adenocarcinoma

**DOI:** 10.18632/aging.202337

**Published:** 2021-01-10

**Authors:** Guan-Chao Ye, Ya-Fei Liu, Lan Huang, Chun-Yang Zhang, Yin-Liang Sheng, Bin Wu, Lu Han, Chun-Li Wu, Bo Dong, Yu Qi

**Affiliations:** 1 Department of Thoracic Surgery, The First Affiliated Hospital of Zhengzhou University, Zhengzhou 450052, China; 2 Biotherapy Center, The First Affiliated Hospital of Zhengzhou University, Zhengzhou 450052, China

**Keywords:** lung adenocarcinoma, differentially expressed genes, differentially expressed miRNAs, miRNA-mRNA regulatory network, PECAM1

## Abstract

In the study, we obtained 36 pairs of lung adenocarcinoma (LUAD) tissues and adjacent non-tumorous tissues. Then, we chose a specific hub-target gene of miRNA and used qRT-PCR to evaluate the expression of PECAM1. We found that the expression level of PECAM1 mRNA in LUAD was significantly lower than that in adjacent nontumor tissues (P<0.0001). Univariate and multivariate analyses were conducted on 481 LUAD patients from The Cancer Genome Atlas (TCGA) according to the Cox proportional hazard regression model to evaluate the impact of PECAM1 expression and other clinicopathological factors on survival. The results showed that the low expression of PECAM1 was an important independent predictor of poor overall survival (HR, 0.704; 95% CI, 0.518-0.957; P = 0.025). Based on the Tumor Immune Estimation Resource (TIMER) database, the relationship between PECAM1 expression and B cell, CD8+ T cell, CD4+ T cell, macrophage, neutrophil, and dendritic cell infiltration was weak in LUAD (P<0.01). In particular, a more significant positive correlation between PECAM1 expression and HLA-complex members, CD1C, NRP1, and ITGAX expression in dendritic cell was detected in LUAD. The mechanism which PECAM1 involved in the development of LUAD may be closely related to changes in the immune microenvironment.

## INTRODUCTION

The increasing incidence of LUAD may be due to increased smoking and air pollution [1]. In addition, LUAD is commonly diagnosed at a late stage, when the cancer has already spread to nearby tissues and metastasized [2]. Late diagnosis and inaccurate prognostication lead to delayed treatment and low survival rates [3]. Therefore, there is an urgent requirement for effective biomarkers to accurately predict the outcomes of LUAD patients in order to provide appropriate adjuvant treatment.

Currently, many studies have found that a class of miRNAs could inhibit the stability and translation of mRNA by binding to specific gene sequences, which be used as potential biomarkers for different types of cancer. Due to the challenges in early diagnosis and prognosis of LUAD, it is important to identify the genetic factors involved in this disease. A variety of bioinformatics tools have been used to evaluate gene expression level, identify unique genes using RNA sequencing and next-generation sequencing data and explore potential significance in the development of tumors [4]. Microarray analysis were widely used to recognize genetic changes in the initiation and development of tumors. In our study, we used mRNA and miRNA microarray data from the Gene Expression Omnibus (GEO) genomics datasets to analyze the differentially expressed genes (DEGs) as well as miRNAs (DEMs). In addition, we hope to provide a potential therapy targets and prognostic markers for LUAD.

Our analysis found that low PECAM1 expression in LUAD is an independent risk factor for poor overall survival (OS). The analysis of TIMER database indicated that the expression of PECAM1 significantly correlated with CD8+ T cells, B cells, macrophages, CD4+ T cells, neutrophils, and dendritic cells infiltration in lung adenocarcinoma. Our study provides potentially prognostic biomarkers with LUAD patients and highlights PECAM1 as a valuable prognostic biomarker in LUAD.

## RESULTS

### Analysis of DEGs and DEMs

**Figure 1:**
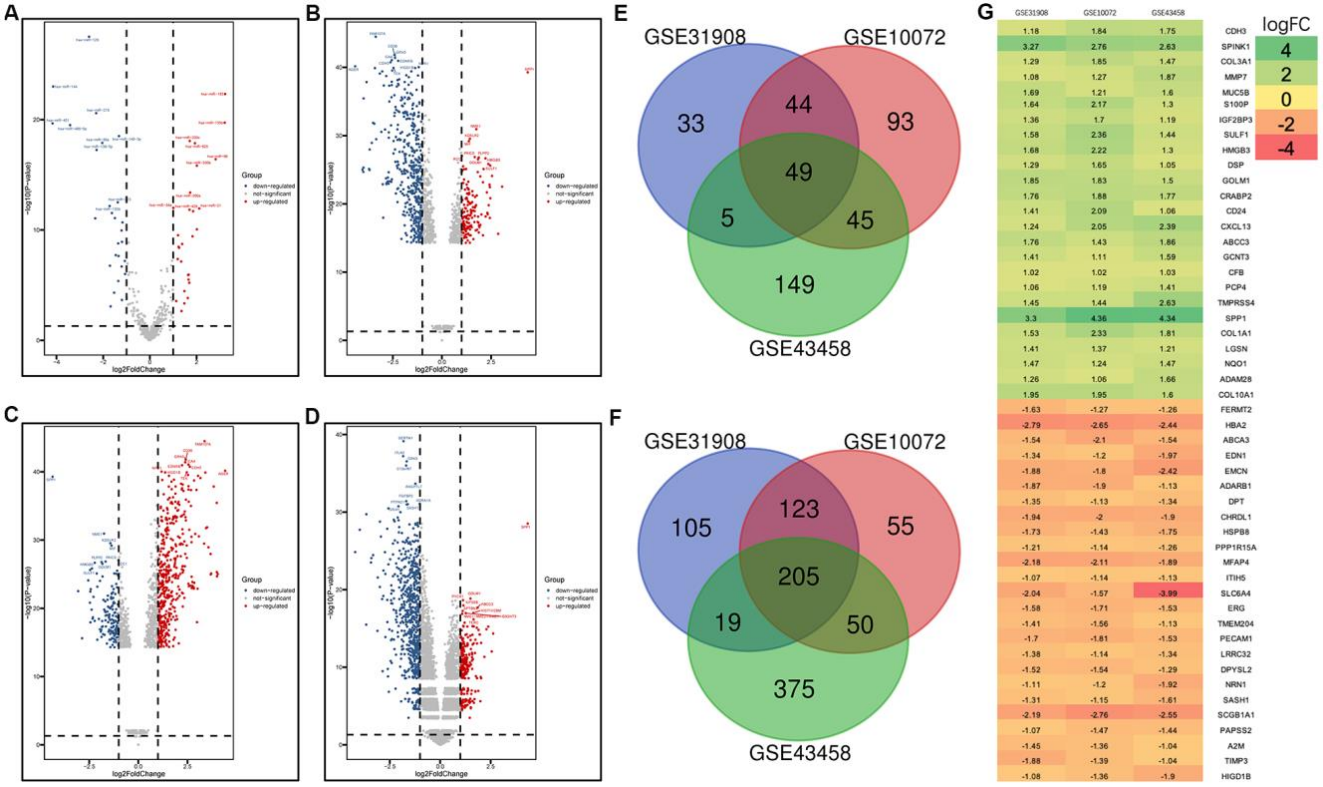
**Volcano plot of gene expression profile data in LUAD and normal samples and the heatmap of the overlapping DEGs.** (**A**) Volcano plot of GSE74190. (**B**) Volcano plot of GSE31908. (**C**) Volcano plot of GSE10072. (**D**) Volcano plot of GSE43458. (**E**) Venn diagram of the upregulated overlapping DEGs. (**F**) Venn diagram of the downregulated overlapping DEGs. (**G**) Heatmap of the overlapping DEGs. Green represents a low fold change (FC) value, and red represents a high fold change FC value. Each column represents one dataset, and each row represents one gene. The number in each rectangle represents the FC in LUAD samples compared with normal samples. The gradual color change from red to green represents the changing process from upregulation to downregulation.

For the GSE74190 dataset, a total of 55 miRNAs (28 upregulated and 27 downregulated miRNAs) were extracted (Figure 1A and Supplementary Table 1), and a total of 711, 597, and 896 genes were extracted from the GSE31908, GSE10072, and GSE43458 datasets, respectively (Figure 1B–1D). These miRNAs and genes had the differently expression between LUAD samples and normal samples. Among the DEGs, 254 overlapped; 49 of these were upregulated (Figure 1E), and 205 were downregulated (Figure 1F). Based on the log fold change (logFC) value, we show the top 25 upregulated and downregulated DEGs as a heat map in Figure 1G.

### The enrichment analysis in overlapping DEGs and DEMs

**Figure 2:**
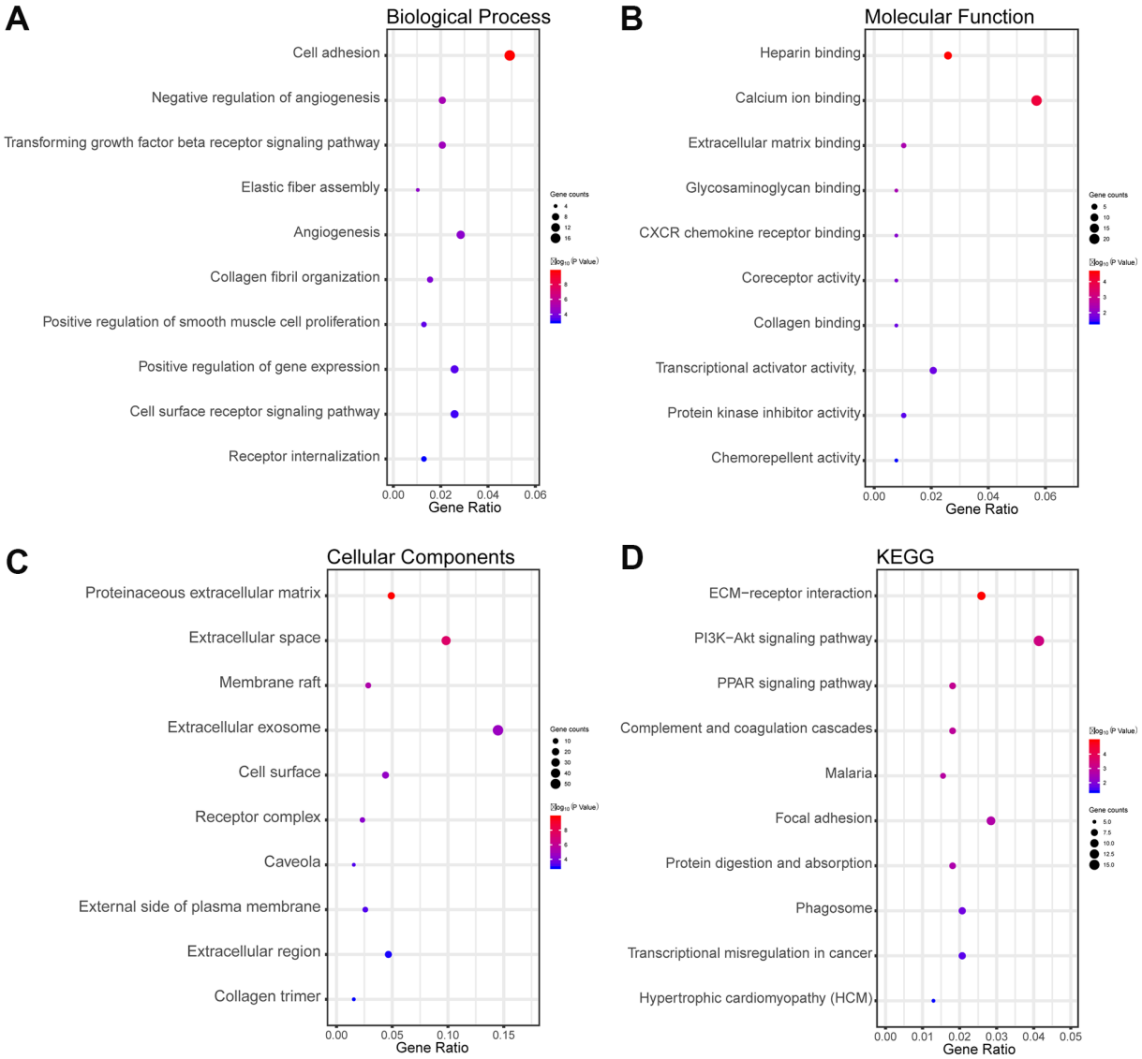
**Functional and pathway enrichment analyses of the overlapping DEGs in LUAD.** (**A**) The BP analysis of DEGs. (**B**) The MF analysis of DEGs. (**C**) The CC analysis of DEGs. (**D**) The KEGG pathway analyses of DEGs. The x-axis represents the q value (−log10), and the y-axis represents the GO term. The GO terms were measured by the rich factor, q value, and number of genes enriched. The greater the rich factor, the greater is the degree of enrichment and the greater the *P* value [0, 1]. The brighter the color of red, the more significant is the term.

The biological processes (BP) results showed that overlapping DEGs were dramatically concentrated in cell adhesion, negative regulation of angiogenesis, and others in Figure 2A. Regarding molecular function (MF), the overlapping differentially expressed genes were significantly concentrated in heparin binding, extracellular matrix (ECM) binding, and others in Figure 2B. Regarding cellular components (CC), the overlapping DEGs were concentrated in proteinaceous ECM, membrane raft, and others in Figure 2C. In addition, the Kyoto Encyclopedia of Genes and Genomes (KEGG) results indicated that the overlapping DEGs were concentrated in pathways involved in ECM-receptor interactions, the PI3K-Akt signaling pathway, and the PPAR signaling pathway in Figure 2D.

**Figure 3:**
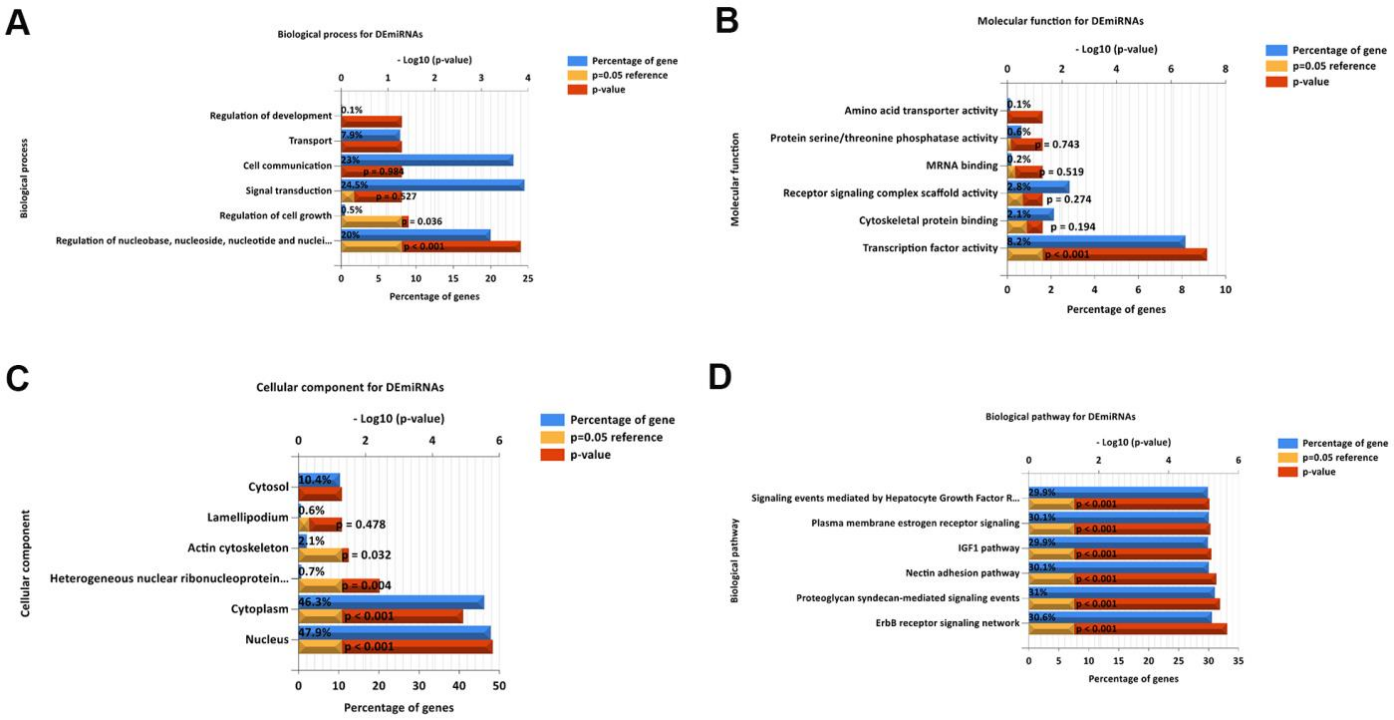
**Functional and pathway enrichment analyses of the DEMs in LUAD.** (**A**) The BP analysis of DEMs. (**B**) the MF analysis of DEMs. (**C**) The CC analysis of DEMs. (**D**) The biological pathway analysis of DEMs. The upper x-axis represents the *P* value (−log10), and the lower x-axis represents the percentage of genes (blue). The y-axis represents the GO term. Yellow represents a *P* = 0.05 as reference, and red represents the specific *P* value. The longer the rectangular zone, the smaller is the *P* value.

To understand thoroughly and fully the biological characteristics of the 55 DEMs in LUAD, we also fulfilled Gene Ontology (GO) annotation and biological pathway analysis. Regarding BP, the DEMs were concentrated in nucleoside, nucleotide, and others in Figure 3A. Regarding MF, the DEMs were significantly enriched in transcription factor activity in Figure 3B. Moreover, regarding CC were nucleus, cytoplasm, heterogeneous nuclear ribonucleoprotein, and actin cytoskeleton in Figure 3C. The most enriched biological pathway terms for the DEMs were related to the ERBB receptor signaling network, nectin adhesion pathway, proteoglycan syndecan-mediated signaling events and others in Figure 3D.

### Construct the protein-protein interaction (PPI) network of the overlapping DEGs

**Figure 4:**
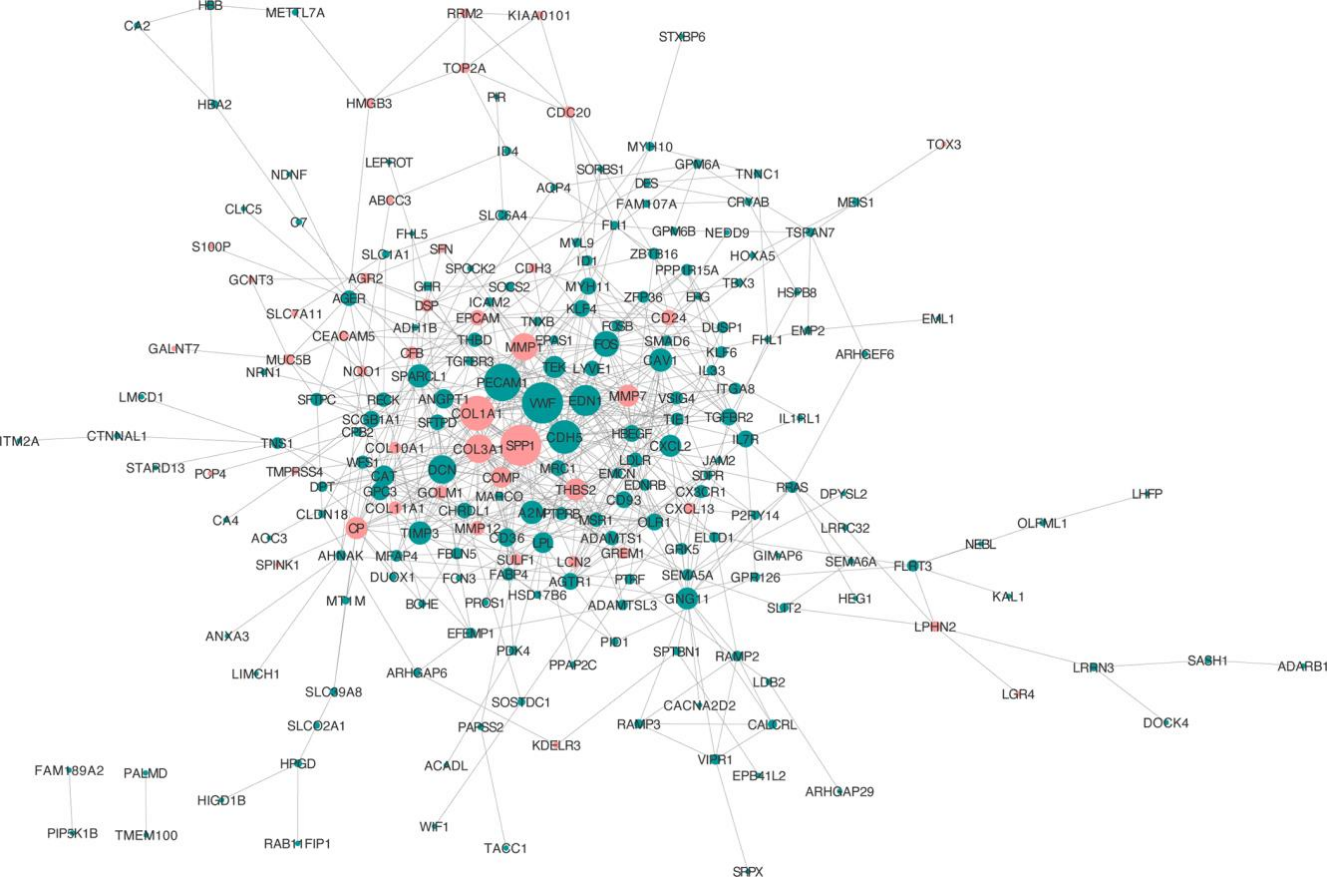
**PPI network analysis of the overlapping DEGs.** Red nodes, upregulated genes; green nodes, downregulated genes.

To assess the interactions of the overlapping DEGs, a PPI network was established in the STRING online website. In total, the network of the DEGs comprises 221 nodes and 607 edges (Figure 4). The more characteristic properties of node degree, the more significant influence the gene has on maintaining the stability of the PPI network. We used Molecular Complex Detection (MCODE) to evaluate functional modules. Eight hub genes were identified with degrees > 14: SPP1 (degree = 29), VWF (degree = 29), PECAM1 (degree = 26), COL1A1 (degree = 24), CDH5 (degree = 23), END1 (degree = 21), COL3A1 (degree = 19), and TEK (degree = 14) (Table 1).

**Table 1:** Hub genes for hypomethylated, highly expressed genes ranked in cytoHubba.

**Catelogy**	**Rank methods in cytoHubba**					
	**MCC**	**MNC**	**Degree**	**EPC**	**Closedness**	**Radiality**
Gene symbol top 15	SPP1	SPP1	SPP1	SPP1	SPP1	PECAM1
	VWF	VWF	VWF	VWF	VWF	SPP1
	PECAM1	PECAM1	PECAM1	COL1A1	PECAM1	VWF
	CDH5	COL1A1	COL1A1	PECAM1	EDN1	EDN1
	TEK	EDN1	CDH5	EDN1	COL1A1	COL1A1
	EDN1	CDH5	EDN1	DCN	CDH5	CDH5
	ANGPT1	COL3A1	DCN	CDH5	DCN	MMP7
	COL1A1	DCN	COL3A1	MMP1	MMP7	DCN
	SPARCL1	FOS	MMP1	COL3A1	MMP1	MMP1
	CP	MMP1	FOS	MMP7	CAV1	CAV1
	GPC3	TEK	TIMP3	COMP	COL3A1	COL3A1
	CHRDL1	THBS2	SPARCL1	THBS2	FOS	FOS
	WFS1	TIMP3	CAV1	TEK	A2M	ANGPT1
	GOLM1	MMP7	A2M	ANGPT1	ANGPT1	TEK
	COL3A1	COMP	TEK	TIMP3	TEK	A2M

### Survival analysis

**Figure 5:**
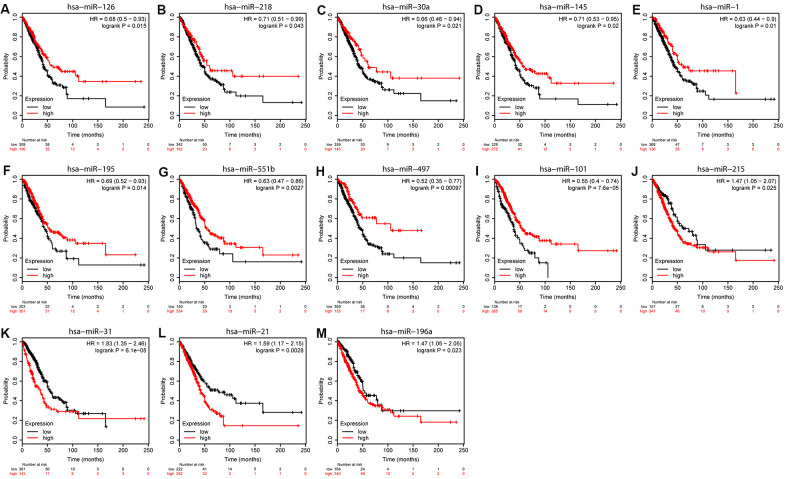
**Overall survival analyses of DEMs.** KM curves depicting OS for LUAD patients with high and low expression of (**A**) hsa-miR-126, (**B**) hsa-miR-218, (**C**) hsa-miR-30a, (**D**) hsa-miR-145, (**E**) hsa-miR-1, (**F**) hsa-miR-195, (**G**) hsa-miR-551b, (**H**) hsa-miR-497, (**I**) hsa-miR-101, (**J**) hsa-miR-215, (**K**) hsa-miR-31, (**L**) hsa-miR-21, and (**M**) hsa-miR-198a.

**Figure 6:**
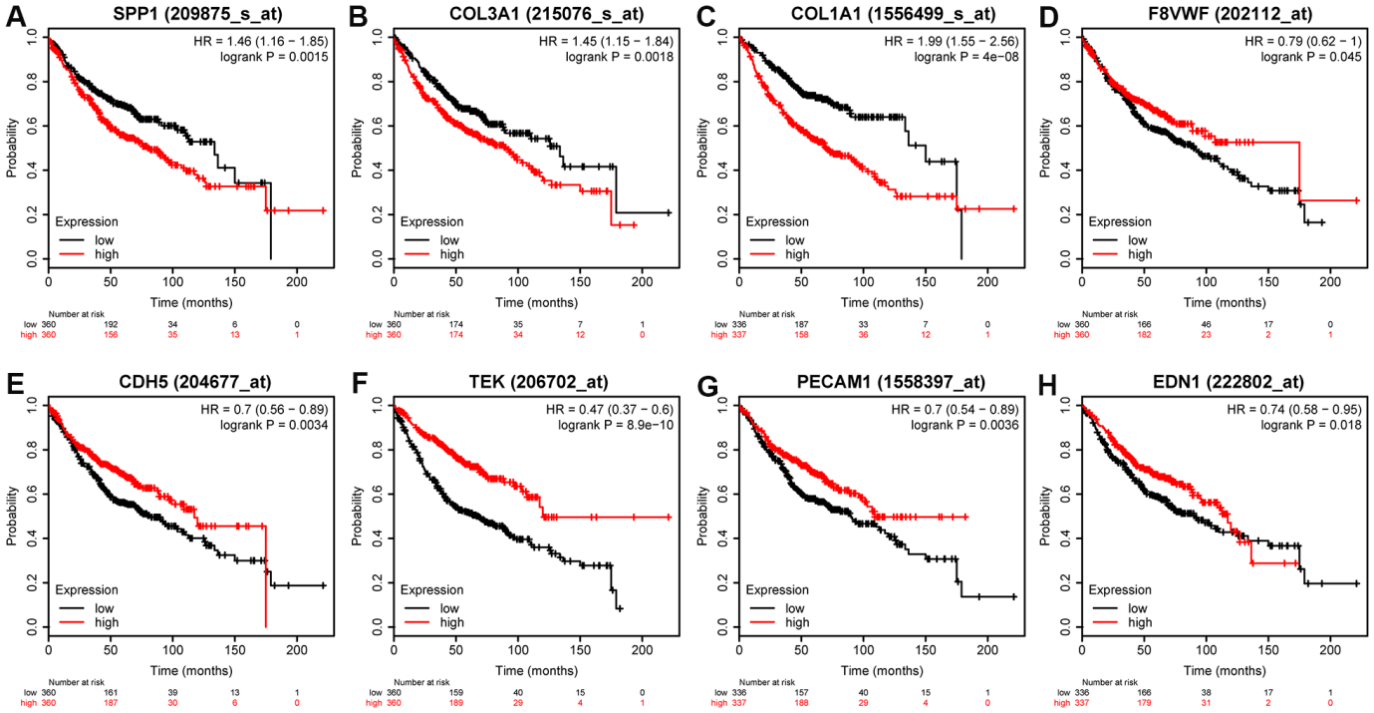
**Prognostic value of eight DEGs in LUAD patients.** Prognostic value of (**A**) SPP1 (log-rank *P* = 0.0015), (**B**) COL3A1 (log-rank *P* = 0.0018), (**C**) OL1A1 (log-rank *P* = 4e-08), (**D**) VWF (log-rank *P* = 0.045), (**E**) CDH5 (log-rank *P* = 0.0034), (**F**) TEK (log-rank *P* = 8.9e-10), (**G**) PECAM1 (log-rank *P* = 0.0036), and (**H**) EDN1 (log-rank *P* = 0.018).

We used Kaplan-Meier (KM) plotter to evaluate the 55 DEMs and 8 hub genes. Among the DEMs, our results showed that low expression level of hsa-miR-126, hsa-miR-218, hsa-miR-30a, hsa-miR-145, hsa-miR-1, hsa-miR-195, hsa-miR-551b, hsa-miR-497, and hsa-miR-101 (*P* < 0.05) was associated with poor OS in LUAD (Figure 5A–5I). In contrast, high expression level of hsa-miR-215, hsa-miR-31, hsa-miR-196a, and hsa-miR-21 (*P* < 0.05) was also associated with poor OS in LUAD (Figure 5J–5M). Regarding the hub genes, high expression level of SPP1, COL1A1, and COL3A1 and low expression level of VWF, PECAM1, EDN1, CDH5, and TEK were associated with the poor OS in LUAD (*P* < 0.05) in Figure 6.

### Transcriptional expression of the hub genes and correlation analysis

**Figure 7:**
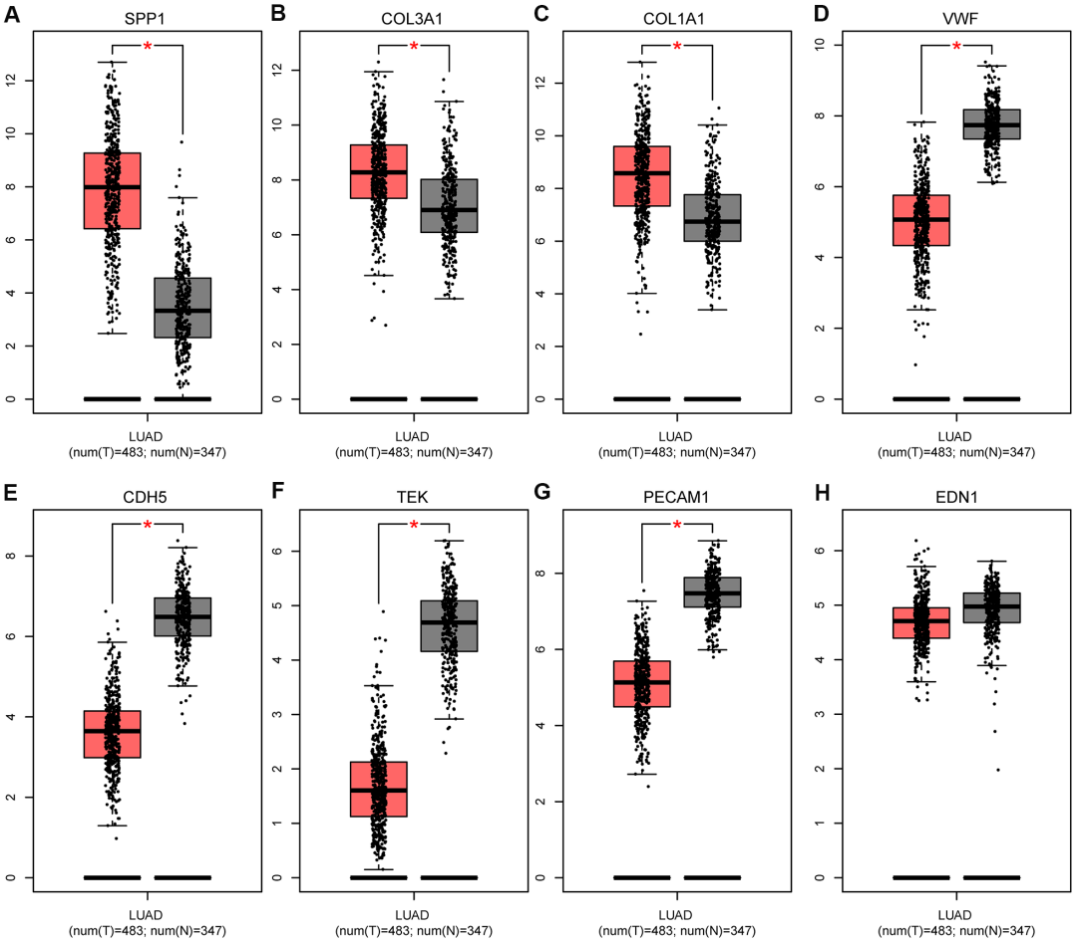
**Expression levels of eight hub genes in human lung adenocarcinoma.** (**A**) SPP1; (**B**) COL3A1; (**C**) COL1A1; (**D**) VWF; (**E**) CDH5; (**F**) TEK; (**G**) PECAM1; and (**H**) EDN1. The gray and red boxes represent normal and cancer tissues, respectively. The expression data are first log_2_(TPM+1) transformed for differential analysis, and the log_2_FC is defined as median (Tumor, T)-median (Normal, N). *P < 0.05, T vs N. Abbreviations: FC, fold change; TPM, transcript per kilobase million.

**Figure 8:**
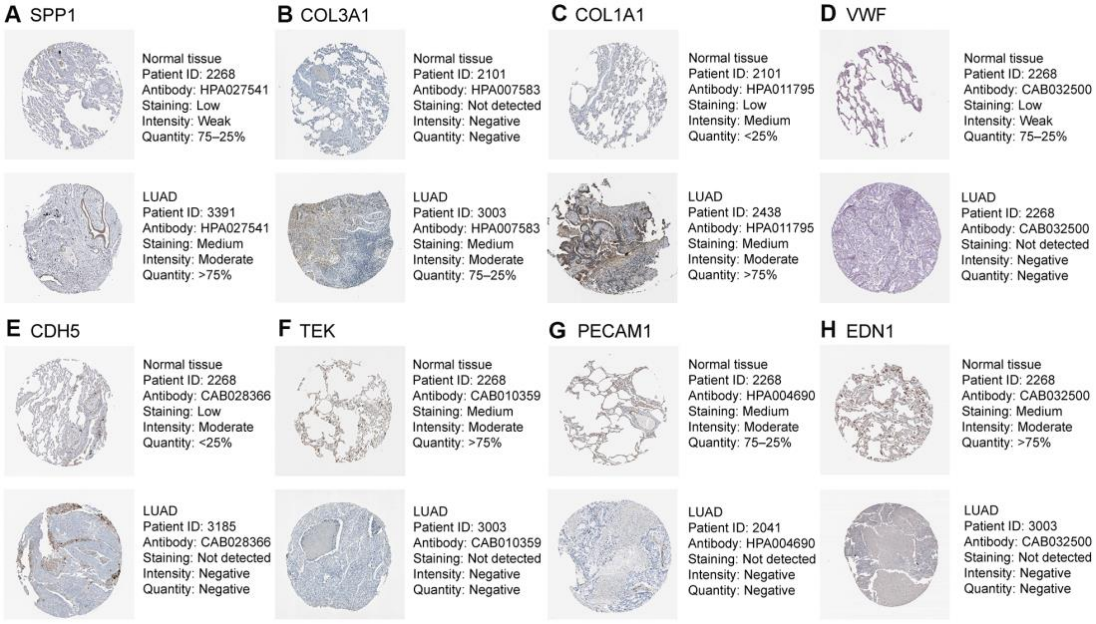
**Validation of eight hub genes using The Human Protein Atlas database.** Expression of (**A**) SPP1, (**B**) COL3A1, (**C**) COL1A1, (**D**) VWF, (**E**) CDH5, (**F**) TEK, (**G**) PECAM1, and (**H**) EDN1.

**Figure 9:**
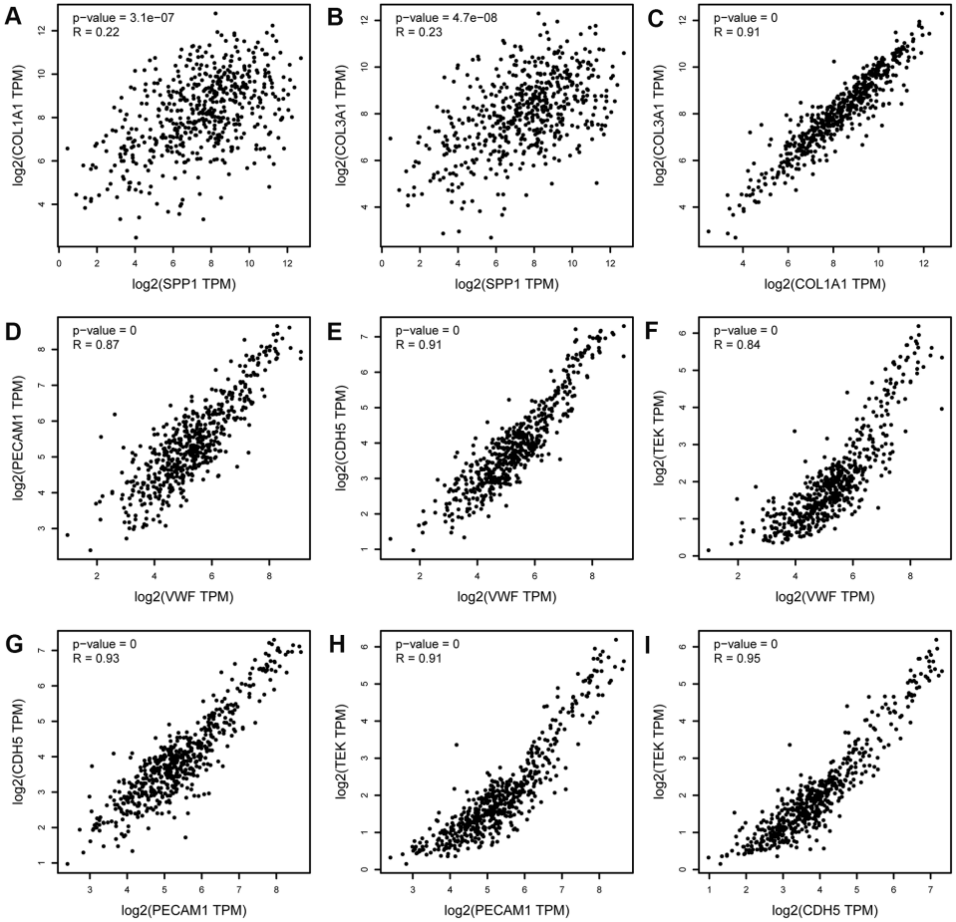
**Correlation analysis of upregulated hub genes (SPP1, COL3A1, and COL1A1) and downregulated hub genes (PECAM1, VWF, CDH5, and TEK) in LUAD.** (**A**) SPP1 and COL1A1, (**B**) SPP1 and COL3A1, (**C**) COL1A1 and COL3A1, (**D**) PECAM1 and VWF, (**E**) PECAM1 and CDH5, (**F**) PECAM1 and TEK, (**G**) VWF and CDH5, (**H**) VWF and TEK, and (**I**) CDH5 and TEK. TPM, transcript per million. The x-axis represents the TPM of the hub gene MYC (log2). The expression levels of upregulated hub genes (SPP1, COL3A1, and COL1A1) were positively correlated with each other. The expression levels of downregulated hub genes (PECAM1, VWF, CDH5, and TEK) were positively correlated with each other.

As shown in Figure 7, based on the Gene Expression Profiling Interactive Analysis (GEPIA) website, mRNA levels were upregulated for three genes and notably downregulated for five genes in LUAD. Moreover, using the Human Protein Atlas (HPA) website, we examined the protein expression level of the hub genes and observed that protein expression of SPP1, COL1A1, and COL3A1 was noticeably upregulated in tumor samples, whereas the protein expression of VWF, PECAM1, EDN1, CDH5, and TEK was noticeably downregulated (Figure 8). In the upregulated hub genes, increased expression of SPP1, COL1A1, and COL3A1 was strongly associated each other in LUAD. In the downregulated hub genes, increased expression in PECAM1, VWF, CDH5, and TEK was strongly associated each other in LUAD (Figure 9).

### Identification of hub genes by ROC analysis

**Figure 10:**
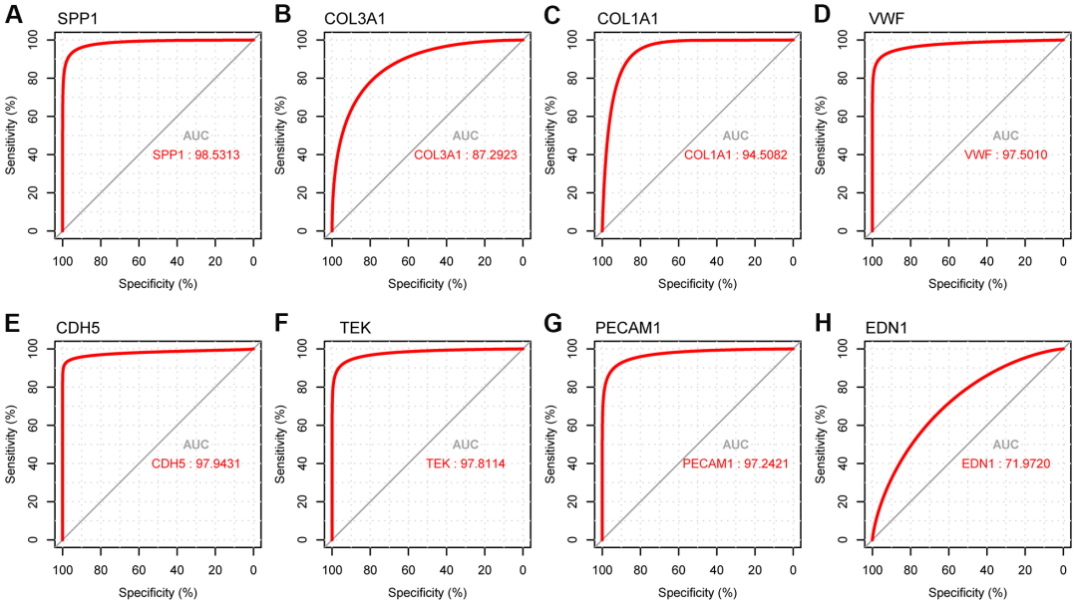
**ROC curve analysis presenting the sensitivity and specificity of hub genes in LUAD diagnosis.** (**A**) ROC curves of SPP1, (**B**) COL3A1, (**C**) COL1A1, (**D**) VWF, (**E**) CDH5, (**F**) TEK, (**G**) PECAM1, and (**H**) END1.

We used the receiver operating characteristic (ROC) curve to predict accuracy of hub genes (Figure 10). The upregulated gene SPP1 and downregulated genes VWF, CDH5, TEK, and PECAM1 had AUC values > 0.97. The ROC curves of the genes had excellent predictive performance and were able to discriminate LUAD from healthy tissues.

### MiRNA–mRNA network

**Figure 11:**
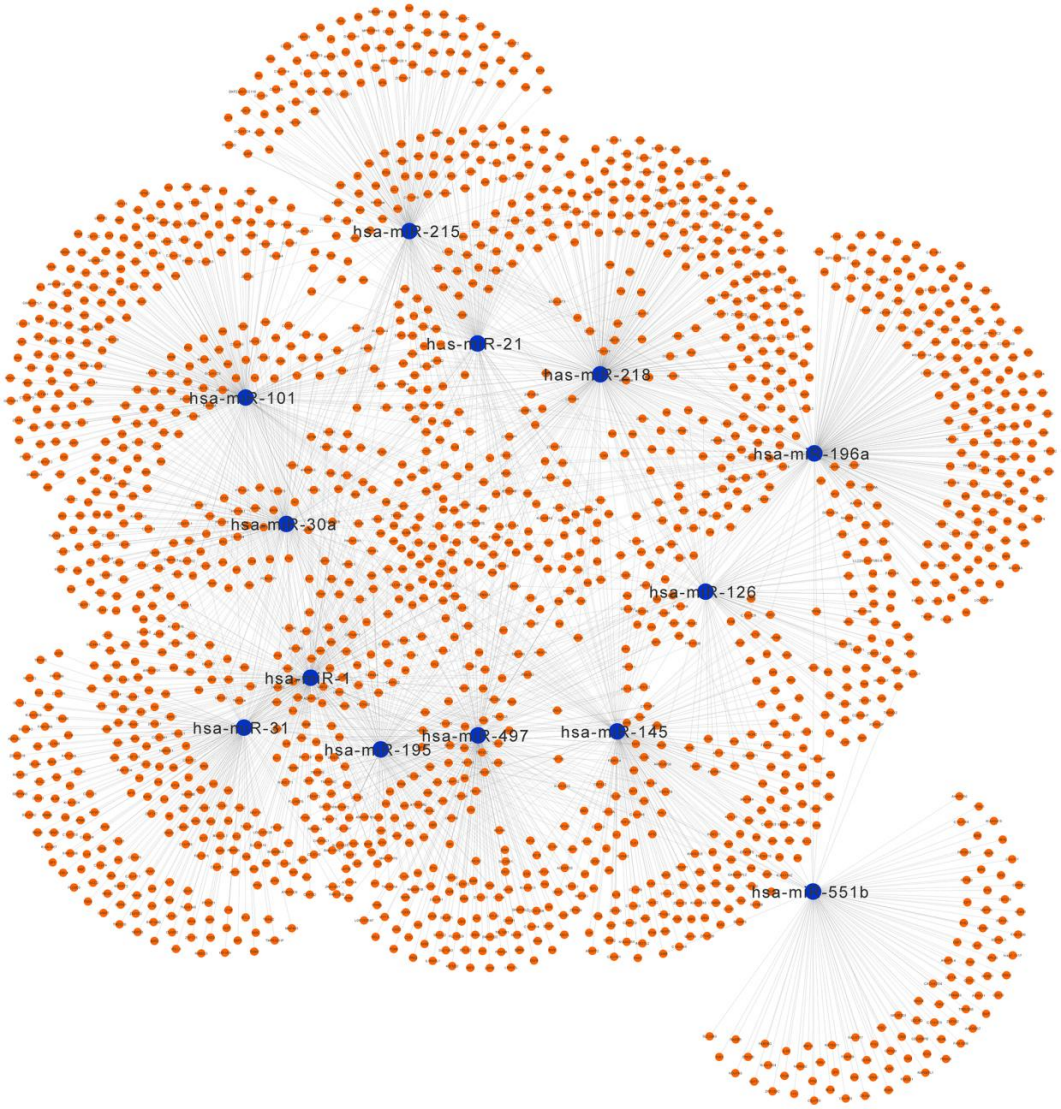
**Regulatory network of the predicted genes and their target miRNAs.** Potential target genes of the significant miRNAs predicted by miRecords.

The network of DEMs and predicted targets is presented in Figure 11. The interactive relationship between hub genes and miRNAs is presented in Supplementary Figure 1 and Supplementary Table 2. Notably, hsa-miR-31 targeted COL1A1, VWF, and TEK; hsa-miR-196a targeted COL1A1 and COL3A1; hsa-miR-215 targeted TEK; hsa-miR-218 targeted COL1A1; hsa-miR-1 targeted EDN1; hsa-miR-145 targeted PECAM1; hsa-miR-195 targeted VWF, COL1A1, and COL3A1; hsa-miR-101 targeted CDH5; and hsa-miR-497 targeted VWF and COL1A1.

### PECAM1 mRNA expression in LUAD tumor and normal samples

**Figure 12:**
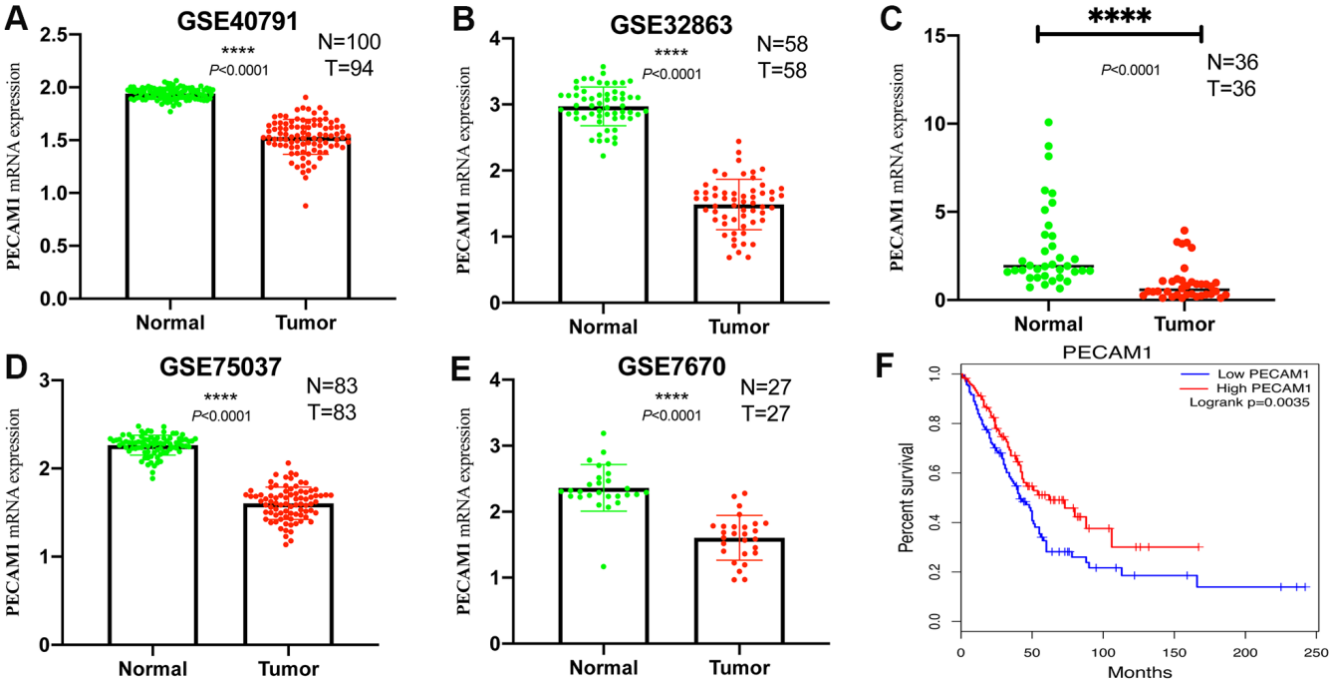
**Comparison of PECAM1 mRNA levels in normal lung tissues and LUAD tissues across four analyses of LUAD.** (**A**–**D**) Each study showed that the PECAM1 mRNA expression levels in normal lung tissues were significantly higher than those in LUAD tissues. (**E**) qRT-PCR analysis of PECAM1 mRNA expression in 47 pairs of LUAD tissues and adjacent nontumor tissues. (**F**) Kaplan-Meier curve of the relationship between PECAM1 mRNA expression and the prognosis of LUAD patients based on the TCGA database.

In four analyses, the mRNA levels of PECAM1 in tumor samples were lower than those in normal samples (*P* < 0.05; Figure 12A–12D). In addition, the qRT-PCR was conducted to demonstrate the transcript level of PECAM1 which was lower in our LUAD tumor samples (*P* <0.0001) in Figure 12E.

### Univariate and multivariate analyses of the impact of PECAM1 expression on survival outcomes

The expression of PECAM1 was significantly related to survival, univariate and multivariate Cox regression model were analyzed to investigate the effect of PECAM1 expression of 481 LUAD patients and other pathologic and clinical variables on survival. Based on univariate analysis, we found that pathological stage (Hazard Ratio (HR), 2.446; 95% Confidence Interval (CI), 1.778-3.363; *P* < 0.001), T stage (HR, 2.295; 95% CI, 1.554-3.388; *P* < 0.001), N stage (HR, 2.468; 95% CI, 1.830-3.328; *P* < 0.001), M stage (HR, 1.926; 95% CI, 1.086-3.416; *P* = 0.025), and PECAM1 expression (HR, 0.634; 95% CI, 0.473-0.874; *P* = 0.005) were the significant factors of survival (Table 2). Moreover, we also analyzed the expression of PECAM1 and other clinicopathological variables (T stage, N stage, and M stage, pathological stage, age) by using multivariate Cox analysis. Above results suggested that the low expression of PECAM1was a significant independent predictor of poor overall survival (HR, 0.704; 95% CI, 0.518-0.957; *P* = 0.025; Table 2). According to the previously clinical follow-up data provided by the TCGA database, patients with the low expression of PECAM1 had poor OS (*P* = 0.0035, Figure 12F).

**Table 2:** Univariate analysis and multivariate analysis of correlation of PECAM1 expression with LUAD patients.

**Clinicopathological features**	**Univariate analysis**			**Multivariate analysis**		
	**HR**	**95% CI**	**P**	**HR**	**95% CI**	**P**
Gender	1.150	0.854-1.549	0.358	0.998	0.735-1.355	0.990
Age(years)	1.199	0.865-1.661	0.276	1.226	0.882-1.705	0.225
T	2.295	1.554-3.388	**0.000**	1.802	1.166-2.786	**0.008**
N	2.468	1.830-3.328	**0.000**	2.011	1.401-2.885	**0.000**
M	1.926	1.086-3.416	**0.025**	1.305	0.684-2.487	0.419
Pathological stage	2.446	1.778-3.363	**0.000**	1.262	0.804-1.983	0.311
PECAM1	0.643	0.473-0.874	**0.005**	0.704	0.518-0.957	**0.025**

Bold values indicate P < 0.05. HR, hazard ratio; CI, confidence interval.

### The PECAM1 expression was correlated to immune cell infiltration in LUAD

**Figure 13:**
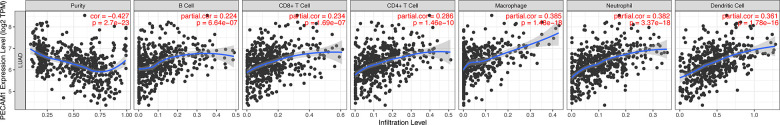
**Correlation of PECAM1 expression with immune cell infiltration levels in LUAD.** Tumor-infiltrating immune cells included B cells, CD4+ T cells, CD8+ T cells, neutrophils, macrophages, and DCs. Gene expression levels against tumor purity are displayed in the left-most panel.

**Figure 14:**
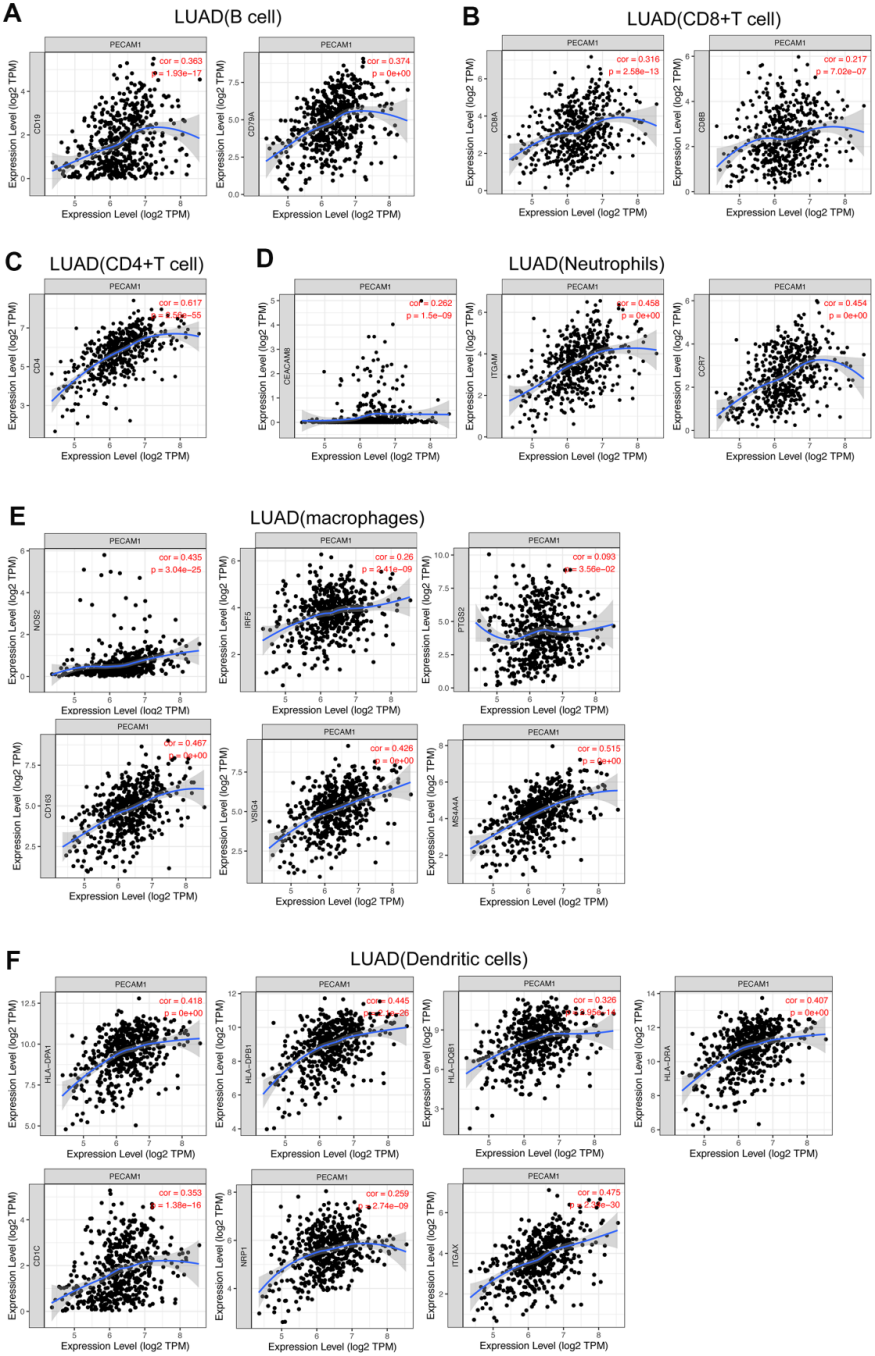
**Correlation of PECAM1 expression with gene markers of tumor-infiltrating immune cells in LUAD.** (**A**) Correlation with gene markers of B cells in LUAD. (**B**) Correlation with gene markers of CD8+ T cells in LUAD. (**C**) Correlation with gene markers of CD4+ T cells in LUAD. (**D**) Correlation with gene markers of neutrophils in LUAD. (**E**) Correlation with gene markers of macrophages in LUAD. (**F**) Correlation with gene markers of DCs in LUAD.

The analysis of TIMER website observed that the expression of PECAM1 was associated with infiltration of several immune cells in LUAD (P < 0.01; Figure 13). Moreover, we investigated the relationship between PECAM1 expression and gene markers of immune cells. The results mentioned above demonstrated that the correlations between the expression of PECAM1 and gene markers of B cells, CD4+ T cells, CD8+ T cells, neutrophils, macrophages, and DCs were determined (Figure 14A–14F). In particular, we detected a positive correlation between the expression of PECAM1 and gene markers of DCs in LUAD (HLA-DPA1: cor = 0.481, P = 0e+∞; HLA-DPB1: cor = 0.445, P = 2.1e-26; HLA-DRA: cor = 0.407, P = 0e+∞; HLA-DQB1: cor = 0.326, P = 2.95e-14; NRP1: cor = 0.259, P = 2.74e-09; ITGAX: cor = 0.475, P = 2.34e-30; CD1C: cor = 0.353, P = 1.38e-16; Figure 14F). Thus, we conjectured that the low expression of PECAM1 might exert influence on anti-tumor immunity in the LUAD microenvironment.

## DISCUSSION

LUAD is a highly malignant tumor that is particularly prone to brain and bone metastasis, which negatively affects patient survival [5]. LUAD is the main cause of cancer death in large part because of the challenges faced in detecting the cancer at an early stage. Lung cancer disproportionately affects the elderly, and management of this complex and lethal disease in older patients is challenging [6]. The senescence and frailty of the elderly adversely affect the management of cancer and patient outcome [7]. Earlier diagnosis and treatment of LUAD may reduce deaths among older patients. Therefore, identifying prognostic markers with high specificity and sensitivity is crucial. Many studies indicated that miRNA and RNA can regulate the expression of genes and involved in many biological processes of human malignant tumors [8]. In particular, recent studies have showed that distinct miRNA and mRNA expression profiles are related to the development of LUAD [9].

In present research, the expression of mRNA and miRNA data downloaded from the GEO database yielded 254 DEGs and 55 DEMs. The enrichment analysis of DEGs were involved in ECM-receptor interaction pathways, the PI3K-Akt signaling pathway, and the PPAR signaling pathway. The DEMs were associated with ERBB receptor signaling network and the IGF1 pathway. Our study and others have shown that lung cancer progression depended not only on the presence of driver mutations within the cancer cells, but also on their interactions with the cellular and ECM environment [10]. Recent evidence suggested that altered metabolic pathways play a crucial role in both cancer occurrence and development, and established link between PPAR signaling, metabolism, and cancer currently represents one of the most active research fields in the literature [11, 12].

We identified eight hub genes 13 and DEMs that were related to the prognosis of patients in LUAD. High expression of four DEMs (miR-215, miR-31, miR-196a, and miR-21) and three hub genes (SPP1, COL1A1, and COL3A1) was associated with worse OS, whereas low expression of nine DEMs (hsa-miR-126, hsa-miR-30a, hsa-miR-218, hsa-miR-1, hsa-miR-145, hsa-miR-195, hsa-miR-551b, hsa-miR-101, and hsa-miR-497) and five hub genes (VWF, PECAM1, EDN1, CDH5, and TEK) was associated with worse OS. The analysis of ROC showed that hub gene levels had excellent predictive performance and were able to discriminate LUAD from healthy tissue. The up-regulated SPP1, COL1A1, and COL3A1 in LUAD were positively correlated with each other, while the down-regulated PECAM1, VWF, EDN1, CDH5, and TEK were in LUAD also positively correlated with each other. Guo et al studied the expression and clinical significance of SPP1 in LUAD and found that SPP1 influenced the initiation and progression of LUAD, which was considered as a new target for molecular treatment [13]. Similarly, we tried to determine the mechanisms of PECAM1 in LUAD. We found that PECAM1 mRNA expression was lower in LUAD. Through using qRT-PCR, it demonstrated that the expression of PECAM1 were down-regulated in tumor tissues. Plus, low PECAM1 expression was significantly associated with clinicopathological features related to development of LUAD. Univariate and multivariate survival analyses identified the low PECAM1 expression was an important independent predictor of unfavorable LUAD prognosis, which demonstrate its potential as a biomarker for LUAD.

In the microenvironment of lung adenocarcinoma, tumor-infiltrating immune cells plays a significant role in cancer progression, which not only inhibit tumor progression by attacking and killing lung cancer cells, but also promote tumor progression by changing the tumor microenvironment [14]. Thus, we evaluated the relationship between PECAM1 expression and infiltration of immune cells. The positive correlation was seen between PECAM1 expression and CD4+ T cells, B cells, CD8+ T cells, neutrophils, macrophages, and DCs. We further analyzed the relationship between PECAM1 expression and molecular markers of tumor-infiltrating immune cells. The present study indicated that the expression of PECAM1 was related to molecular markers of B cells, CD4+ T cells, CD8+ T cells, neutrophils, macrophages, and dendritic cells. Especially, PECAM1 expression was significantly correlated with HLA-complex member (ITGAX, CD1C, NRP1) in dendritic cells. Due to DCs are vital antigen-presenting cells (APCs) triggering T-cell–mediated immune response [15], Thus, the functional impairment of dendritic cells could influence the body’s anti-tumor immunity reaction. While fundamental studies should be established causally, the result indicated that low expression of PECAM1 could compromise DC-mediated antitumor immune response in LUAD.

Dysregulated miRNAs have shown promise as noninvasive diagnostic and prognostic biomarkers for LUAD. Numerous studies indicated that altered expression of miRNAs is related to various cancers, including lung cancer [16]. In our study, high expression of four DEMs (has-miR-31, has-miR-21, has-miR-196a, and has-miR-215) and low expression of nine DEMs (hsa-miR-126, hsa-miR-218, hsa-miR-30a, hsa-miR-145, hsa-miR-1, hsa-miR-195, hsa-miR-551b, hsa-miR-497, and hsa-miR-101) were associated with worse OS in LUAD. Liu et al found that miR-126-3p was crucial in the invasion of NSCLC by targeting CCR1 [17]. Shi et al determined that downregulation of miR-218 was noted to serve an essential role in epithelial-mesenchymal transition (EMT) and cancer metastasis of lung cancer by targeting Slug/ZEB2 signaling [18]. MiR-30a played a crucial role in the occurrence of NSCLC [19]. Liu et al reported that miR-145 was downregulated in tumor tissues and that miR-145 overexpression resulted in downregulation of N-cadherin, vimentin, and E-cadherin, suggesting decreased EMT activity [20]. Korde et al found that levels of miR-1 were lower in NSCLC than normal tissue and that overexpression of miR-1 reduced tumor growth and angiogenesis [21]. Liang et al found that downregulating miR-195 simultaneously stimulated cell proliferation and inhibited cell apoptosis in LUAD [22]. Lin et al identified miR-551b as a cancer-specific miRNA that may predict the progression and prognosis of LUAD [23]. Cui et al indicated that inhibition of miR-101 promotes progression of NSCLC through activation of the Wnt/β-catenin signaling pathway [24]. Yin et al reported that miR-145 and miR-497 were downregulated in lung cancer cell lines and that miR-145 and miR-497 overexpression repressed TGF-β-induced EMT and the migration and invasion of cancer cell [25]. Recent researches have showed that miR-31 modulates the NSCLC cell cycle by targeting hMLH1 [26]. Bao et al revealed that serum miR-196a-5p may be a valuable noninvasive biomarker for the clinical diagnosis of NSCLC [27]. In our study, all miRNAs which associated with prognosis have been reported previously in lung cancer except miR-215. And maybe they could be the useful biomarker predictive of prognosis in LUAD.

PECAM1 can code for CD31, belonging to the immunoglobulin superfamily of adhesion molecule, which can maintain and restore vascular integrity by the wingless-related integration site signaling pathway [28]. Yu et al analyzed the related microarray datasets (GSE9804) and identified that PECAM1 played a crucial role in the tumorigenesis of LUAD via regulation of vascular endothelial growth factor (VEGF) expression [29]. The mRNA transcription and the protein expression levels, we found that PECAM1 expression was significant down-regulated in LUAD tumor tissues. Importantly, we found that low expression of PECAM1 was a significant independent predictor of poor OS by univariate and multivariate survival analyses in LUAD. SPP1 has also been demonstrated in mice and humans to affect tumor cell migration, adhesion, and invasion, indicating its possible use as a diagnostic biomarker and potential therapeutic target in cancer [30]. Our enrichment analysis indicated that SPP1 is enriched in cell adhesion, the PI3K-Akt signaling pathway, and ECM-receptor interactions, which play an important role in LUAD. In addition, we confirmed that COL1A1 expression was higher in LUAD tissues. COL1A1 was reported to be enriched in various biological processes such as cell proliferation, metastasis, invasion, and angiogenesis [31]. Wei et al analyzed the GSE75037 microarray dataset and determined that COL1A1, CTGF, COL1A2, COL3A1, and BGN were involved in cell adhesion, the PI3K-Akt signaling pathway, and ECM-receptor interaction [32]. Above study was consistent with our findings. Besides its essential role in hemostasis, there is growing evidence that VWF has antitumor effects, mainly related to angiogenesis and apoptosis [33]. EDN1 were implicated in cancer progression, apoptosis, EMT, stromal reaction, and tumor invasion, spread, and drug resistance [34]. CDH5 is an adhesion molecule and member of the selectin family that contributes significantly to tumorigenesis and tumor progression [35]. TEK is a receptor tyrosine kinase that is expressed in endothelial cells. Dysregulated TEK expression has also been observed in several cancer, indicating that TEK expression may have prognostic value in these cancers [36].

In sum, these data supported that DEMs and hub genes may have clinical utility as prognostic markers in LUAD. Although there are some limitations in the current research, the combination of multichip analysis and bioinformatics analysis has significant advantages in identifying potential diagnostic biomarkers and therapeutic targets of cancers.

Therefore, the present research provided the potential prognosis biomarkers as well as future therapeutic targets for LUAD and indicated that PECAM1, in particular, may be a novel biomarker of survival that provided a novel diagnostic biomarkers and therapeutic targets for the treatment of LUAD and may help to improve the therapeutic efforts in the future. However, further studies were needed to explore the molecular mechanisms and biological functions of these microRNAs and hub genes and to evaluate whether they can serve as biomarkers or therapeutic targets in LUAD patients.

## MATERIALS AND METHODS

### LUAD dataset preprocessing

We downloaded GSE31908, GSE10072, and GSE43458 datasets and GSE74190 miRNA expression data from GEO by searching using the following key terms: (“microRNA” OR “miRNA” OR “mRNA”) AND (“Lung adenocarcinoma” OR “LUAD”). The GSE31908 dataset included 20 normal lung tissues and 30 LUAD tissues. GSE10072 comprised 107 samples, including 49 normal samples and 58 tumors. GSE43458 contained 110 samples, including 80 LUAD tissues and 30 normal tissues. GSE74190 comprised 80 samples, including 36 tumors and 44 normal tissues.

### Data processing

The GEO2R could compare different groups in GEO datasets and identify DEGs and DEMs. Adjusted *P* < 0.05 and an absolute logFC > 1 for the DEGs and the DEMs were used as the cutoff criteria. The Venn plots were used to show the overlapping DEGs that were up or down-regulated in the GSE31908, GSE10072, and GSE43458 datasets.

### Clinical specimen collection

In total, we obtained 36 patients of LUAD tissues and corresponding normal tissues from the First Affiliated Hospital of Zhengzhou University between April 2019 and January 2020. These patient samples had never received chemoradiotherapy prior to surgical resection. Written informed consents were received from all patients, and this study was approved by the Ethics Committee of the First Affiliated Hospital of Zhengzhou University.

### The enrichment analysis in overlapping DEGs and DEMs

Database for Annotation, Visualization, and Integrated Discovery (DAVID) has integrated many biological data analytical tools. We visualized the key BP, MF, CC and KEGG pathways of DEGs by using the DAVID. Similarly, we also performed a functional enrichment analysis of the DEMs by FunRich, which is also primarily used for the functional enrichment analysis of genes and proteins. Visualization of GO terms and KEGG results was performed by bubble diagrams and bar charts.

### Construct the PPI network of the overlapping DEGs

With setting an interaction score ≥0.4, we analyzed the PPI network of DEGs by STRING website. Then, we downloaded the network relationship file, and the top 15 genes were identified in accordance with Cytoscape 3.7.1 and MCODE, an app of Cytoscape software (MCC, MNC, Degree, EPC, Closeness, Radiality ranking of MCODE). We used the intersection of these sets to obtain the final hub genes.

### The survival analysis of DEMs and hub genes

Respectively, KM plotter was used to perform an OS analysis for DEMs and hub genes in 513 and 866 LUAD patient samples. Based on the gene transcriptional expression of a given gene, we separated patients into high and low groups and performed KM plots by the KM plotter. In addition, the chart showed the HR with the 95% CI and the log-rank *P* value, and the number at risk is displayed below the curves.

### The expression and correlation analysis of the hub genes

GEPIA, a well-developed interactive online server applied to a standard processing pipeline, including 9736 tumor and 8587 normal tissues mRNA data. We used GEPIA to appraise the mRNA expression levels of hub genes in LUAD and normal tissues. A correlation analysis presents pairwise genes using the Pearson, Spearman, and Kendall correlation statistics based on GTEx and/or TCGA expression data in GEPIA.

### ROC analysis

The ROC curves were carried out to assess the specificity of the DEGs for LUAD diagnosis. The ROC curves were plotter and the AUC values were calculated using the ROC package for R.

### Construct the miRNA–mRNA network

We used the miRecords database to predict the target genes of DEMs using 11 established algorithms. Only target genes of DEMs predicted by at least three prediction databases were accepted. Next, the regulatory network of the predicted genes and miRNAs that targeted them were visualized in Cytoscape.

### Verification of PECAM1 by the GEO database

By using the Mann-Whitney U test, we validated mRNA expression levels of PECAM1 between LUAD tissues and normal tissues in GEO databases. The details of GEO databases are as follows: GSE40791, GSE32863, GSE75037, and GSE7670. Above datasets were obtained from the GEO datasets. The preprocessing of each dataset adopts the preprocessing results of the original author.

### Verification of PECAM1 expression by qRT-PCR

Total RNAs were isolated from LUAD tissues using TriZol reagent (TakaRa, Japan). Then total RNAs was reverse-transcribed using the PrimeScript RT reagent Kit with gDNA Eraser (TakaRa, Japan). PECAM1 expression was validated by qRT-PCR using SYBR Premix Ex Taq II (TakaRa, Japan) on the LightCycler 480 II Real-Time PCR System (Roche, Basel, Switzerland). Glyceraldehyde 3-phosphate dehydrogenase (GAPDH) was amplified as an internal control. The primer sequences (sunya Biotech, zhengzhou, China) were as follows:

PECAM1 forward:TTCACCAAGATAGCCTCAAAGTCG

PECAM1 reverse:TGGGAGAGCATTTCACATACGACT

GAPDH forward: GGAGCGAGATCCCTCCAAAAT

GAPDH reverse: GGCTGTTGTCATACTTCTCATGG

### Univariate and multivariate cox regression analyses

The Cox regression models were used to calculate univariate and multivariate analysis. The factors that had a significant effect on survival were identified. We calculated the value of the clinicopathological factors and PECAM1 expression on survival by the univariate and multivariate analysis. Firstly, we downloaded mRNA expression and clinical data from TCGA. Then we screened the whole survival and gene expression data. Finally, the data of 481 patients were obtained, which were analyzed by Cox regression models. The median expression was used as a cutoff value, the patients at the top 50% expression level of PECAM1 were assigned to the high group and the other patients were assigned to the low group. We used R software’s survival package to analyze and visualize the data.

### TIMER analysis

The online tool TIMER is a resource for comprehensive analysis of immune infiltration across multiple cancer types. We used TIMER on LUAD sample data to assess these infiltrates. Then, the relationship between expression of PECAM1 and immune infiltration was demonstrated. Furthermore, the database was used to explore the association between PECAM1 expression and gene markers of immune cells in LUAD.

### Statistical analysis

We used the Mann-Whitney U test to test the differential expression of PECAM1 in tumor and corresponding normal samples. Univariate and multivariate survival analysis were carried out by using the Cox proportional hazards regression models. All statistical analyses were used by IBM SPSS statistics (version 26.0) and R software (version 4.0.2). *P* < 0.05 was considered statistically significant.

## Supplementary Material

Supplementary Figure 1

Supplementary Tables
