# Supplementary material for: Key microRNAs and hub genes associated with poor prognosis in lung adenocarcinoma
Source: Aging (Albany NY). 2021 Jan 10;13(3):3742–62. doi: 10.18632/aging.202337 (PMC7906143; doi:10.18632/aging.202337)
Supplement: Supplementary Figure 1 [file aging-13-202337-s001.pdf]

SUPPLEMENTARY FIGURE

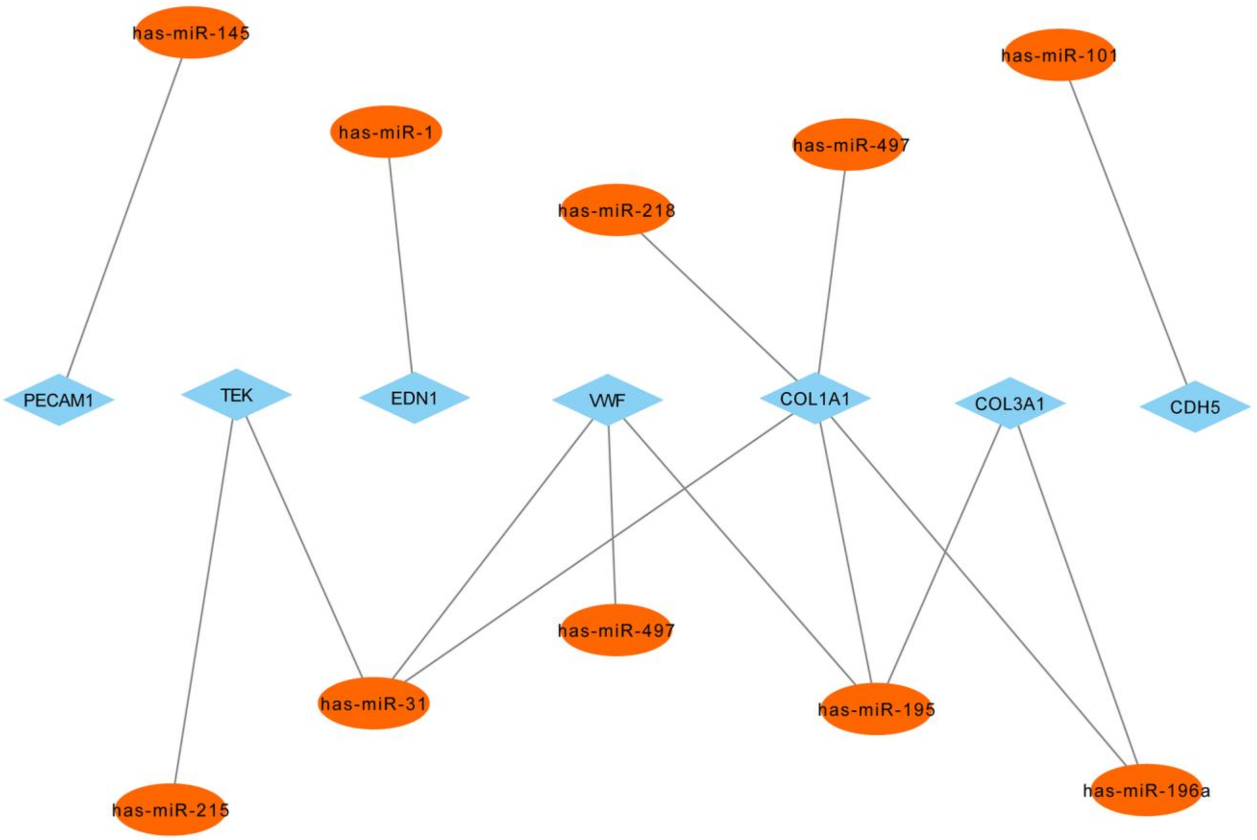

Supplementary Figure 1. The miRNA–hub gene network of the significant DEMs and their hub genes. Red, DEMs; blue, hub genes.
