# Supplementary material for: Key microRNAs and hub genes associated with poor prognosis in lung adenocarcinoma
Source: Aging (Albany NY). 2021 Jan 10;13(3):3742–62. doi: 10.18632/aging.202337 (PMC7906143; doi:10.18632/aging.202337)
Supplement: Supplementary Tables [file aging-13-202337-s002.pdf]

SUPPLEMENTARY TABLES

Supplementary Table 1. The up/down-regulated DEMs in GSE74190.

Up/down	DEmiRNAs	*adj.P.Val*	*P-value*	*logFC*
Up-regulated	hsa-miR-183	9.66E-21	4.70E-23	3.223301
	hsa-miR-135b	2.56E-18	1.89E-20	3.20692
	hsa-miR-200c	6.97E-17	8.49E-19	1.707107
	hsa-miR-625	9.21E-17	1.35E-18	1.93122
	hsa-miR-96	2.25E-15	3.84E-17	2.818292
	hsa-miR-200b	8.12E-15	1.48E-16	2.012788
	hsa-miR-200a	2.09E-12	4.07E-14	1.726916
	hsa-miR-34a	3.92E-11	1.00E-12	1.005749
	hsa-miR-21	4.17E-11	1.12E-12	2.114328
	hsa-miR-429	5.22E-11	1.46E-12	1.697122
	hsa-miR-182	6.82E-11	1.99E-12	1.852576
	hsa-miR-130b	2.39E-09	8.72E-11	2.014196
	hsa-miR-425	7.41E-09	2.98E-10	1.208185
	hsa-miR-210	9.07E-09	3.76E-10	1.826569
	hsa-miR-148a	3.93E-08	1.87E-09	1.383262
	hsa-miR-141	6.87E-08	3.35E-09	1.232262
	hsa-miR-542-3p	7.97E-08	4.07E-09	1.251343
	hsa-miR-450a	7.74E-07	4.34E-08	1.192052
	hsa-miR-424	1.21E-06	7.19E-08	1.342517
	hsa-miR-224	1.45E-05	1.13E-06	1.661966
	hsa-miR-375	1.48E-05	1.19E-06	1.636325
	hsa-miR-196a	3.80E-05	3.15E-06	1.656606
	hsa-miR-7	7.13E-05	6.08E-06	1.688777
	hsa-miR-31	3.46E-04	3.46E-05	1.478019
	hsa-miR-192	1.32E-03	1.47E-04	1.623764
	hsa-miR-127-3p	2.35E-03	2.72E-04	1.128916
	hsa-miR-215	3.68E-03	4.48E-04	1.476747
	hsa-miR-194	1.42E-02	2.22E-03	1.355854
Down-regulated	hsa-miR-126	2.51E-25	3.05E-28	-2.606823
	hsa-miR-144	4.12E-21	1.00E-23	-4.144946
	hsa-miR-218	4.23E-19	2.58E-21	-2.302513
	hsa-miR-451	2.56E-18	2.18E-20	-4.1685
	hsa-miR-486-5p	3.19E-18	3.11E-20	-3.417527
	hsa-miR-140-3p	2.83E-17	3.10E-19	-1.32449
	hsa-miR-30a	9.21E-17	1.28E-18	-2.039716
	hsa-miR-139-5p	3.51E-16	5.55E-18	-2.283491
	hsa-miR-223	1.35E-11	3.11E-13	-1.375561
	hsa-miR-133b	2.77E-11	6.74E-13	-1.724604
	hsa-miR-498	9.59E-11	2.92E-12	-1.626849
	hsa-miR-145	2.62E-10	8.29E-12	-1.498766
	hsa-miR-338-3p	2.66E-10	8.75E-12	-2.341491
	hsa-miR-497	5.83E-10	2.06E-11	-1.173224
	hsa-miR-195	2.93E-09	1.11E-10	-1.213904
	hsa-miR-101	2.82E-08	1.20E-09	-1.192292
	hsa-miR-143	3.56E-08	1.65E-09	-1.357178
	hsa-miR-638	3.53E-07	1.89E-08	-1.459486

hsa-miR-134	4.20E-07	2.30E-08	-1.344233
hsa-miR-1225-5p	1.11E-06	6.47E-08	-1.074196
hsa-miR-1	2.68E-06	1.73E-07	-1.710784
hsa-miR-557	3.12E-06	2.05E-07	-1.208886
hsa-miR-30b	1.28E-05	9.81E-07	-1.063555
hsa-miR-572	4.99E-05	4.20E-06	-1.352622
hsa-miR-34b	4.52E-04	4.79E-05	-1.530313
hsa-miR-551b	1.94E-03	2.20E-04	-1.196408
hsa-miR-34c-5p	6.41E-03	8.35E-04	-1.682751

Supplementary Table 2. The interactive relationship between hub genes and miRNAs.

Hub genes	DEmiRNAs
SPP1	NA
COL1A1	has-miR-31, has-miR-196a, has-miR-218, has-miR-195, has-miR-497
COL3A1	has-miR-196a, has-miR-195
VWF	has-miR-497, has-miR-195, has-miR-31
PECAM1	has-miR-145
EDN1	has-miR-1
CDH5	has-miR-101
TEK	has-miR-31, has-miR-215
